# Clinical Evolution of Neuropsychiatric Symptoms in Alzheimer's Disease and Dementia With Lewy Bodies in a Post‐Mortem Cohort

**DOI:** 10.1002/gps.70084

**Published:** 2025-04-28

**Authors:** Lucy L. Gibson, Ragnhild Eide Skogseth, Tibor Hortobagyi, Audun Osland Vik‐Mo, Clive Ballard, Dag Aarsland

**Affiliations:** ^1^ Department of Psychological Medicine King's College London Centre of Healthy Brain Ageing Institute of Psychiatry, Psychology, and Neuroscience London UK; ^2^ Department of Geriatric Medicine Haraldsplass Deaconess Hospital Bergen Norway; ^3^ Department of Clinical Sciences Faculty of Medicine University of Bergen Bergen Norway; ^4^ Department of Neurology University of Debrecen Debrecen Hungary; ^5^ Institute of Neuropathology University Hospital Zurich Zurich Switzerland; ^6^ Centre for Age‐Related Medicine Stavanger University Hospital Stavanger Norway; ^7^ Department of Clinical Medicine University of Bergen Bergen Norway; ^8^ Medical School (HB) University of Exeter Exeter UK

**Keywords:** dementia with Lewy bodies, neuropathology, neuropsychiatric symptoms

## Abstract

**Background:**

Almost all patients with neurodegenerative dementias experience neuropsychiatric symptoms (NPS) but the timing and clinical course is highly variable.

**Methods:**

In a prospective cohort study in Western Norway, patients with a new diagnosis of mild dementia were assessed annually in the Neuropsychiatric Inventory (NPI) for up to 9 years until death. Patients with *post‐mortem* neuropathological diagnoses of Alzheimer's disease (pAD) (*n* = 37), Lewy body disease (pLBD) (*n* = 14) or meeting criteria for both AD and LBD (mixed AD+LBD) (*n* = 11) were included in this study. Neuropathological assessment was performed according to standardised protocols and blind to clinical information. In mixed effects logistic regression, longitudinal change in NPS was explored across neuropathological diagnoses and substrates. Additionally, the odds of NPS early and late in disease was evaluated in logistic regression.

**Results:**

Early onset hallucinations were significantly more common in pLBD than pAD (OR 0.069 [95% CI 0.012–0.397], *p* = 0.003) or mixed AD+LBD (OR 0.09 [95% CI 0.010–0.771], *p* = 0.028) and there was a greater increase in the odds of hallucinations over time in pAD and AD+LBD than pLBD such that there was was no difference in the prevalence of late‐onset hallucinations between pLBD, pAD or AD+LBD. Hallucinations early in disease were associated with higher LBD α‐synuclein stages and neocortical LBD, in addition and sparser amyloid distribution. Higher density of amyloid plaques, tau tangles, cerebrovascular disease and increasing additional co‐pathologies were associated with increasing odds of hallucinations over time.

**Conclusions:**

LBD, without significant comorbid AD pathology, is associated with hallucinations early in the course of disease while multiple other pathologies may be implicated in aetiology of late‐onset hallucinations. Hallucinations increase in AD+LBD as disease progresses, a trajectory more closely aligned with AD than LBD.

**Summary**

- Significant Outcomes
  ◦ Early‐onset hallucinations are associated with Lewy body disease (LBD).
  ◦ In Alzheimer's disease (AD) and mixed AD‐LBD, the odds of developing hallucinations increase as the disease progresses.
  ◦ Multiple co‐pathologies are associated with increased odds of late‐onset hallucinations.
- Limitations
  ◦ The sample size of our included cohort is small and may be underpowered to detect other differences between the neuropathological groups.
  ◦ Only end‐point pathological changes can be identified in this post‐mortem study and in vivo biomarkers are needed to characterise pathological changes earlier in disease.

## 1 Introduction

Neuropsychiatric symptoms (NPS) are common and disabling symptoms which affect almost all patients with neurodegenerative dementia at some point in the course of disease [1]. However, there is significant heterogeneity in the prevalence and evolution of NPS between different patients both inter and intra‐diagnostically [2, 3]. Currently, the mechanisms driving NPS in neurodegenerative dementias are poorly understood and there are few available treatment options for patients. Clinical studies suggest psychosis is most common in dementia with Lewy bodies (DLB) [2] such that the presence of psychosis in Alzheimer's disease (AD) is associated with increased likelihood of misdiagnosis as DLB [4]. Longitudinal studies with post‐mortem confirmation of diagnosis are needed to characterise the course of NPS, improve diagnostic accuracy and optimise treatment of these important symptoms.

Lewy bodies, formed from aggregates of misfolded α‐synuclein, are the neuropathological hallmark of Lewy body disease (LBD) but they are rarely an isolated pathology in DLB [5, 6]. Co‐morbid Alzheimer's disease neuropathological change (ADNC) is found in more than 50% of post‐mortem cases of LBD, with clinical implications including accelerated cognitive decline and poor prognosis [7, 8, 9]. Clinicopathological studies suggest there may also be implications for NPS; agitation, depression and psychosis have been associated with increased propagation of tau and neurofibrillary tangles in both AD and DLB, and co‐pathologies are reported to contribute additively to the burden of NPS [10, 11, 12, 13]. However, most clinicopathological studies of NPS have been cross‐sectional, and longitudinal studies are needed to explore the influence of neuropathological changes on the evolution of NPS from disease onset.

The diagnosis of DLB remains challenging in clinical practice and is often associated with long delays, more frequent clinical contacts and initial misdiagnoses [14, 15]. LBD is identified in more than 20% of cases with dementia in neuropathological studies but only 5% of dementia patients receive a clinical diagnosis of DLB cases and the presence of co‐morbid AD pathology reduces the diagnostic accuracy [16, 17, 18, 19]. The advent of potentially disease modifying therapies targeting specific neuropathological changes has increased the importance of accurately identifying co‐pathology to facilitate a precision medicine approach [20, 21]. Better characterisation of the clinical presentation and natural course of symptoms associated with neuropathological change is needed. In mixed AD+LBD, while cognitive decline is accelerated and core features of DLB may be less prominent, the neuropsychiatric phenotype is unclear [19, 22, 23]. Several clinicopathological studies have suggested co‐pathologies may contribute to greater burden of NPS [10, 11, 24], while in vivo studies with biomarkers suggest AD co‐pathology may be associated with less frequent visual hallucinations in DLB [23, 25, 26, 27]. In AD, hallucinations are associated with a longer duration of illness [28, 29] suggesting this co‐pathology may contribute to NPS late in the disease course. Given psychotic symptoms are reported more frequently in LBD than in AD [2], it may be that symptoms early in the course of disease are better able to differentiate between underlying pathology where mixed pathologies are less prevalent [29, 30].

In a longitudinal clinicopathological study with annual ante‐mortem clinical assessments, we aimed to explore associations between neuropathological change and the evolution of NPS from diagnosis of dementia. Previous clinical studies in this cohort have illustrated the prevalence and high heterogeneity in the longitudinal course of NPS across patients but this has not yet been corroborated with neuropathological analysis [2, 31]. Post‐mortem analysis of this cohort has demonstrated good concordance between clinical and neuropathological diagnoses [27] but in this study, we also aimed to identify differences in the clinical course of NPS in patients with mixed AD+LBD pathology relative to patients with more pure LBD and AD.

## 2 Materials and Methods

### 2.1 Study Design

The Dementia Study of Western Norway (DemVest) is a longitudinal cohort study with annual assessment of patients referred to dementia clinics in Hordaland and Rogaland counties. Recruitment procedures have been described previously [32], but briefly, referrals were requested from general practices in the area in addition to all dementia diagnostic clinics. Patients were initially recruited between 2005 and 2007 but patients with LBD were selectively recruited until 2013 to enhance numbers in this group. Inclusion criteria were diagnosis of mild dementia according to DSM IV criteria with Mini‐Mental State Examination (MMSE) score ≥ 20 and/or Clinical Dementia Rating score ≤ 1, no acute delirium, terminal illness, major somatic or psychiatric illness with effects on cognition.

The study was approved by the regional committee for medical and health research ethics in western Norway (REK 2010/633). As the patients all had mild dementia at inclusion, they were able to provide written informed consent, including consent to autopsy.

The original study recruited 265 patients, 21 withdrew and 2 were found not to have dementia at follow up [27]. Of these patients, 70 came to autopsy by June 2023. Seven were excluded due to a primary neuropathological diagnosis other than AD or LBD (frontotemporal dementia *n* = 2, vascular dementia *n* = 3, progressive supranuclear palsy [*n* = 1], normal ageing [*n* = 1]). One outlier patient with a duration of symptoms of 10 years at the study baseline was excluded (≥ 3SD than the mean duration of symptoms in the included cohort 2.81 ± 2.39 years).

### 2.2 Clinical Evaluation

Baseline evaluation was conducted by specialists in geriatric medicine or psychiatry and supported by a research nurse. In addition to a semi‐structured interview of patients and caregivers, general clinical and neurological examinations, a neuropsychological examination including the MMSE, the Neuropsychiatric Inventory (NPI), plasma sampling and MRIs were performed [27, 32] A subsample also consented to lumbar puncture and/or autopsy. Patients were followed up annually until death with a mean follow up time of 4.94 years (2.0 SD).

Clinical diagnoses of dementia were made according to DSM IV criteria in addition to the NINCDS ADRDA (National Institute of Neurological and Communicative Diseases and Stroke/Alzheimer's Disease and Related Disorders Association) for AD [33], the McKeith 2005 criteria for DLB [34] and the Movement Disorder Society (MDS) criteria for PDD [35]. Clinical diagnoses were reviewed by a consensus group of three specialists taking into account all available information, including the electronic medical records. Diagnoses were reevaluated after 5 years and upon completion of the autopsy cohort based on all available information except neuropathology.

### 2.3 Assessment of Neuropsychiatric Symptoms

Neuropsychiatric symptoms were assessed in the interview with family or caregivers with the validated Norwegian 12‐question NPI [36, 37]. The NPI‐Nursing Home was used when patients moved to nursing homes. When NPS were reported, items were rated by frequency (1–4) and severity (1–3), multiplied to give an overall score. For each symptom, a previously established cut‐off score ≥ 4 was used to determine the presence of clinically significant symptoms [2, 38].

The total score in the NPI was also included, with a total score ≥ 36 used as a cut‐off to indicate significant burden of NPS, similar to previous studies [2, 39]. To assess differences in the clinical course of NPS, the presence of early (occurring at the baseline or first follow up assessment) and late symptoms (reported at, or after, the 2 year follow up assessment) were evaluated.

### 2.4 Neuropathological Examination

#### 2.4.1 Sampling and Characterization of Postmortem Brain Samples

The neuropathological assessment of post‐mortem samples has been previously described [27]. Standard protocols were followed for brain dissection, macroscopic description, regional sampling, tissue processing and staining [40, 41, 42]. Procedures for histological and immunohistochemical studies were performed in accordance with published guidelines, as reported previously [27, 40, 41, 42, 43] and, each case was reviewed by an experienced neuropathologist blinded to clinical data. For DLB, the pathological diagnosis was made according to international consensus criteria for DLB, classified as LBD if the likelihood of a DLB syndrome according to McKeith et al. was ‘intermediate’ or ‘high’ [34]. The Braak staging of LBD and location of LBD were additionally reported [44]. For ADNC, neurofibrillary tangles were scored according to Braak stage [45, 46] and amyloid‐β pathology was scored using CERAD‐NP for the density of neuritic plaques (none, sparse, moderate or frequent) [47].

A neuropathological diagnosis of AD was made in patients with a NIA Reagan classification of ‘high’ [48], in addition to at least CERAD moderate amyloid plaque pathology and Braak tau stage ≥ IV. Patients were classified according to the primary neuropathological diagnosis applied by an experienced neuropathologist blinded to clinical data and patients meeting AD and LBD criteria were classified as mixed AD+LBD. Patients were classified as pure AD (pAD) if moderate or severe ADNC was present in the absence of intermediate LBD in the McKeith criteria described above while patients were classified as pure LBD (pLBD) if there was intermediate likelihood of LBD in the absence of moderate or severe ADNC. Vascular co‐pathology (cerebral amyloid angiopathy, small vessel disease, infarcts) was assessed and classified by severity (mild, moderate or severe) [49]. The presence of limbic‐predominant age‐related TDP‐43 encephalopathy (LATE‐NC) was also staged according to guidelines; classified as stage 1 if TDP‐43 deposits were found in the amygdala, stage 2 if TDP‐43 deposits were in the amygdala and hippocampus and stage 3 if deposits were in the middle frontal gyrus [50]. The overall number of pathologies at post‐mortem for each patient was also calculated with binary cut‐offs for each pathology (vascular disease: at least moderate severity, TDP‐43 LATE NC: ≥ stage 2, LBD: intermediate or severe LBD, beta‐amyloid: at least moderate CERAD staging and tau tangles: Braak stages ≥ IV).

### 2.5 Statistics

Demographic and clinical variables were compared across patients characterised by neuropathological diagnosis (pAD, pLBD and mixed AD+LBD). Comparisons were made using Fisher exact tests for categorical variables, and Kruskal‐Wallis tests for non‐parametric continuous variables. The presence of clinically significant hallucinations was chosen as the primary outcome, both due to its clinical relevance and potential as a discriminatory marker between neuropathological diagnoses [2, 51]. Presence of clinically significant delusions, depression, agitation, apathy and overall NPI score were secondary outcomes. Despite the importance of REM sleep behaviour disorder (RBD) as a marker for DLB, the night‐time behaviour item on the NPI was not included as an outcome because it has not been validated to detect RBD in DLB and some of its features characterising circadian rhythm disruption (early morning wakening, nocturnal awakenings, insomnia) may be more common in AD. [52, 53, 54, 55].

To compare the clinical course of NPS between neuropathological diagnoses, we used multilevel mixed effects logistic regression models with a random intercept and slope for each participant to account for individual variability in longitudinal analyses and the correlation of repeated measures over time. There was a high degree of missingness after year 5 of follow up (55% mortality year 6; 79% year 7% and 84% mortality year 9), with some variation in survival time across neuropathological groups (see Table 1). To minimise the impact of this, only the first 5 years of follow up were included in the mixed effects regression models. Presence of clinically significant hallucinations was the primary outcome variable with neuropathological diagnosis and the interaction with time as the predictor variables. The model was adjusted for potential confounders such as age, sex, MMSE as an index of clinical severity and survival time to adjust for differences to the time of death from each assessment. Mixed effect logistic regression models were also used to explore longitudinal change in delusions, depression, agitation, apathy and clinically significant total NPS.

**TABLE 1:** Demographic and clinical variables by neuropathological diagnosis.

	Total *n* = 62	AD *n* = 37	LBD *n* = 14	AD+LBD *n* = 11	*p*
Age, *y* (SD)	74.0 (7.8)	73.6 (8.3)	72.7 (7.7)	77 (5.5)	0.394
Female (%)	33 (53.2)	23 (62.2)	4 (28.6)	6 (54.6)	0.108
Symptom duration at baseline, *y* (SD)	2.8 (2.4)	2.3 (1.5)	3.2 (2.2)	2.9 (2.2)	0.452
Education, *y* (SD)	9.9 (3.3)	9.8 (3.3)	11.1 (3.9)	8.6 (1.9)	0.228
Time to death from baseline, *y* (SD)	6.77 (3.1)	7.34 (2.8)	6.01 (3.6)	5.84 (3.1)	0.134
Interval from last assessment to death, *y* (SD)	1.54 (1.6)	1.63 (1.8)	1.65 (1.5)	1.11 (1.2)	0.279
Baseline MMSE (SD)	24.5 (2.5)	24.3 (2.3)	25.1 (2.2)	24.2 (3.4)	0.561
Other co‐pathology					
TDP‐43 LATE NC					0.781
None	42 (67.7)	26 (70.3)	10 (71.4)	6 (54.6)	
Stage 1	4 (6.5)	2 (5.4)	1 (7.1)	1 (9.1)	
Stage 2	11 (17.7)	6 (16.2)	3 (21.4)	2 (18.2)	
Stage 3	5 (8.1)	3 (8.1)	0	2 (18.2)	
Vascular co‐pathology	48 (77.4	31 (83.8)	7 (50.0)	10 (90.9)	0.010
None	6 (9.7)	2 (5.4)	4 (28.6)	0	
Mild	9 (14.5)	5 (13.5)	3 (21.4)	1 (9.1)	
Moderate	18 (29.0)	7 (18.9)	5 (35.7)	6 (54.6)	
Severe	29 (46.8)	23 (62.2)	2 (14.3)	4 (36.4)	
Presence of clinically significant NPS at any time during follow up, *n* (%)					
Delusions	29 (46.8)	18 (48.7)	7 (50)	4 (36.4)	0.775
Hallucinations	33 (53.2)	17 (42)	10 (71.4)	6 (54)	0.255
Depression	41 (66.1)	25 (67.6)	7 (50)	9 (81.8)	0.271
Apathy	50 (80.7)	30 (81.1)	11 (78.6)	9 (81.8)	1.00
Agitation	30 (48.4)	16 (43.2)	7 (50)	7 (63.6)	0.463
NPI > 36	38 (61.3)	24 (64.9)	8 (57.1)	6 (54.6)	0.816
‘Early’ NPS: Present at baseline or year 1 assessment, *n* (%)					
Delusions	16 (25.8)	10 (27.0)	4 (28.6)	2 (18.2)	0.848
Hallucinations	15 (24.2)	4 (10.8)	9 (64.3)	2 (18.2)	**<** **0.001**
Depression	22 (35.5)	13 (35.1)	2 (14.3)	6 (54.6)	0.112
Apathy	28 (45.2)	15 (40.5)	9 (64.3)	4 (36.4)	0.270
Agitation	14 (22.6)	7 (18.9)	3 (21.4)	1 (9.1)	0.807
NPI ≥ 36	18 (29.0)	10 (27.0)	6 (42.9)	2 (18.2)	0.429
‘Late’ NPS: Present at follow up ≥ 2, *n* (%)					
Delusions	20 (35.1)	14 (40)	4 (33.3)	2 (20)	0.540
Hallucinations	27 (46.6)	15 (42.9)	7 (53.4)	5 (50)	0.759
Depression	28 (51.9)	18 (52.9)	5 (45.5)	6 (66.7)	0.630
Apathy	44 (75.9)	29 (82.9)	7 (53.9)	8 (80)	0.145
Agitation	20 (37.0)	10 (29.4)	5 (45.5)	5 (55.6)	0.285
NPI ≥ 36	26 (44.8)	18 (51.4)	4 (30.8)	4 (40)	0.432

*Note:* NPS are assessed as present if a clinically significant symptom is reported as present at any time during follow up. The bold values signify *p* values < 0.05.

Abbreviations: MMSE; mini‐mental state examination, NPI; neuropsychiatric inventory, TDP‐43 LATE NC; Limbic predominant age‐related TDP‐43 encephalopathy neuropathologic change.

Presence of hallucinations was assessed early (at baseline or first follow up assessment) and late (≥ 2 years assessment). The risk of early and late hallucinations was compared across neuropathological diagnoses in multinomial logistic regression, with results presented as Odds Ratios (OR). The analyses were adjusted for adjusted for age, sex, MMSE score (clinical severity index) and time to death (defined as time from baseline for early hallucinations and the interval between the last assessment and death for late hallucinations) Additionally, the neuropathological correlates of patients with early and late hallucinations were described and the risk of hallucinations associated with each neuropathological substrate was assessed in logistic regression adjusted for age, sex, time to death and MMSE with results presented as Odds Ratios (OR). Additional mixed effect logistic regression models with clinically significant hallucinations as the outcome and individual neuropathological substrates as the predictor variables were used to explore associations with longitudinal change in hallucinations, adjusted for age, sex, time to death and MMSE score. All analyses were conducted in STATA 16.1.

## 3 Results

### 3.1 Summary and Overall Presence of NPI

A total of 62 patients were included, *n* = 14 with a neuropathological diagnosis of pLBD, *n* = 37 with pAD and *n* = 11 meeting criteria for both LBD and AD (mixed AD+LBD). There were no demographic differences across the patients characterised by neuropathological diagnosis (see Table 1). Almost all patients had significant NPS (scoring ≥ 4 in the NPI item) at some time during follow up and apathy was the commonest symptom reported. No differences were found in the frequency of symptoms reported overall during longitudinal follow up between the neuropathological groups. However, hallucinations were significantly more common in early disease in pLBD than pAD or mixed AD+LBD.

### 3.2 Concordance With Clinical Data

Neuropathological diagnoses of pLBD were 93% concordant (agreement in *n* = 13/14) with clinical diagnoses of DLB (*n* = 8) or PDD (*n* = 5) both at baseline and in the final diagnosis, expanding on the cohort reported in an earlier study [27]. At the final clinical assessment one patient was diagnosed with probable AD and 8 with probable DLB and 5 with PDD. Neuropathological diagnoses of pAD matched the clinical diagnosis in 89% (*n* = 33) of cases at the final assessment, other diagnoses included Vascular dementia [VaD] (*n* = 1), mixed AD and VaD (*n* = 2), and mild cognitive impairment (*n* = 1). In patients with mixed AD+LBD, at baseline 6 were diagnosed with AD and 5 with probable DLB, and at the final assessment 2 with AD were reclassified as DLB (see Table 2).

**TABLE 2:** Concordance between clinical and neuropathological diagnoses.

	Baseline clinical diagnosis				Final clinical diagnosis			
	AD (possible/probable)	DLB/PDD (possible/probable)	Mixed (VaD+AD)	Other	AD (possible/probable)	DLB/PDD (possible/probable)	Mixed (VaD+AD)	Other
AD	30	3	0	4	33	0	2	2
DLB	1	13	0	0	1	13	0	0
AD+LBD	6	5	0	0	4	7	0	0

### 3.3 Early‐Onset and Late‐Onset Hallucinations

#### 3.3.1 Associations Across Neuropathological Diagnoses

**FIGURE 1:**
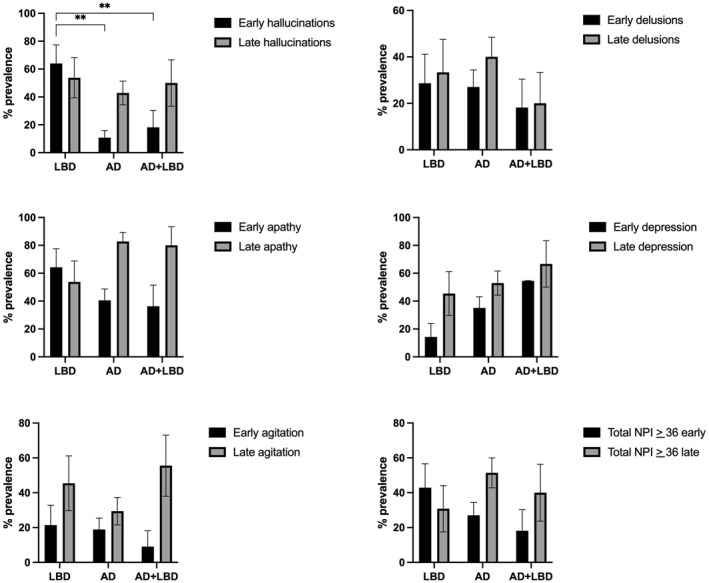
Prevalence of NPS in pure LBD, pure AD and mixed AD+LBD. **p < 0.001.

**FIGURE 2:**
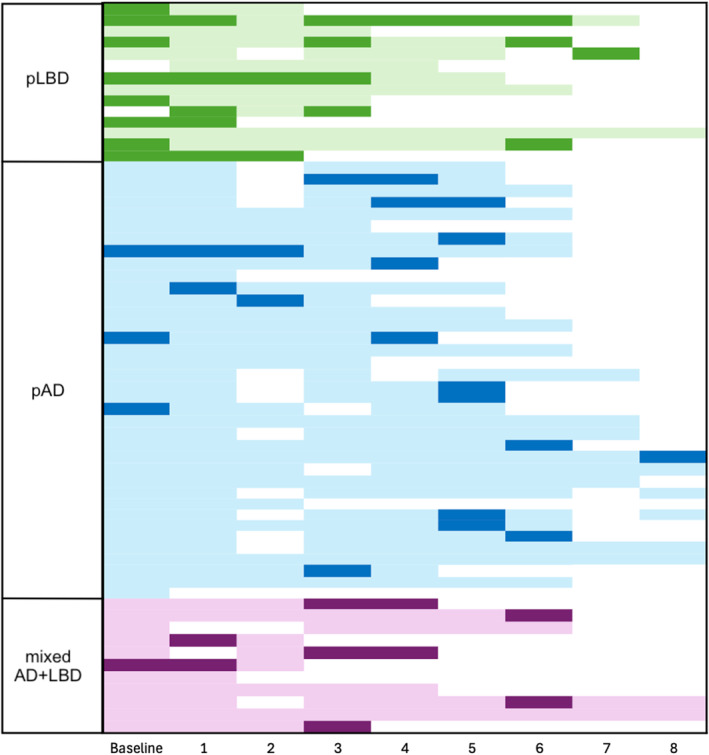
Longitudinal presence of hallucinations for each patient is shown in each row progressing over time up to 8 years of follow up and grouped by neuropathological diagnosis (pLBD = green; blue = pAD; purple mixed AD+LBD). The shaded dark indicates presence of hallucinations at each time point, pale indicates absence, and empty cells indicate no testing at this time point.

The prevalence of clinically significant symptoms early and late in the course of disease is described in Table 1 and illustrated in Figure 1. In logistic regression adjusted for age, sex, time to death and cognition, early hallucinations were significantly more common in pLBD than pAD (OR 0.069 [95% CI 0.012–0.397], *p* = 0.003) or mixed AD+LBD (OR 0.09 [95% CI 0.010–0.771], *p* = 0.028) shown in Figure 1. There was no difference in the frequency of late hallucinations across neuropathological diagnoses. The clinical course of hallucinations over time is illustrated for each patient grouped by neuropathological diagnosis in Figure 2.

### 3.4 Clinical Course of Neuropsychiatric Symptoms Across Diagnoses

**FIGURE 3:**
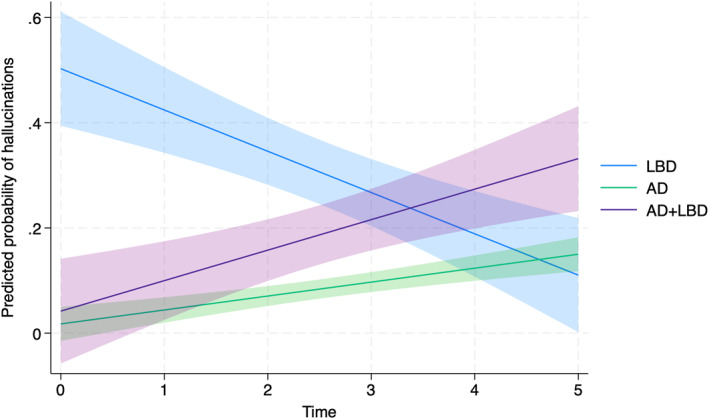
Predicted probability of hallucinations over time by neuropathological diagnosis.

In mixed effect logistic regression model adjusted for age, sex and time to death, the odds of hallucinations at baseline were significantly greater in pLBD than pAD (*β* = −4.94, 95% CI −8.35 to −1.53, *p* = 0.005) and mixed AD+LBD (*β* = −4.18, 95% CI −8.08 to −0.28, *p* = 0.036). However, there was a significantly greater increase in the incidence of hallucinations over time in both pAD and mixed AD+LBD relative to pLBD (pAD *β* = 1.38, 95% CI 0.34 to 2.42, *p* = 0.009; mixed AD+LBD *β* = 1.35 95% CI 0.07 to 2.63, *p* = 0.039). These differences in the predicted probability of hallucinations over time between neuropathological groups are shown in Figure 3. If the models were also adjusted for cognition, at baseline hallucinations remained significantly more common in pLBD than pAD or mixed AD+LBD with significantly greater increase in hallucinations over time in pAD relative to pLBD (see Supporting Information S1: Table 1). However, the greater increase in hallucinations over time in mixed AD+LBD relative to pLBD was no longer significant (*β* = 1.55 95% CI 0.07 to 2.63, *p* = 0.068) suggesting the increase in hallucinations is not independent of cognition later in the course of disease.

There was no difference in the frequency or longitudinal change in delusions, apathy, agitation, symptoms of depression or total NPI score across neuropathological diagnoses (see Supporting Information S1: Table 1).

#### 3.4.1 Neuropathological Description of Patients With Hallucinations Early and Late in the Clinical Course

**FIGURE 4:**
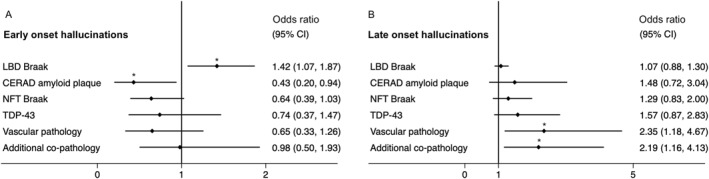
(A and B). Odds ratio of early (3A) and late hallucinations (3B) for each neuropathological substrate in logistic regression adjusted for age, sex, time to death and cognition.

A description of the neuropathological characteristics of patients with hallucinations early and late in disease is included in Table 3. In logistic regression adjusted for age, sex, time to death and cognition higher LBD Braak α‐synuclein stages were associated with increased odds of early‐onset hallucinations (OR 1.42 [95% CI 1.07–1.87], *p* = 0.014) and LBD in the neocortical (but not brainstem or limbic regions) were associated with increased odds of early‐onset hallucinations (OR 6.88 [95% CI 1.18–40.3], *p* = 0.032). Higher CERAD amyloid plaque stages were associated with lower odds of early‐onset hallucinations (CERAD amyloid OR 0.43 [95% CI 0.20–0.94], *p* = 0.035) (see Figure 4A).

**TABLE 3:** Neuropathological characteristics of cohort separated by the presence of early and late hallucinations.

	Total *n* = 62	Early hallucinations				Late hallucinations		
		Present (*n* = 15)	Absent (*n* = 47)		*p*	Present (*n* = 27)	Absent (*n* = 31)	*p*
NIA Reagan AD likelihood					0.004			1.00
None	1 (1.6)	0		1 (2.1)		0	1 (3.2)	
Low	3 (4.8)	2 (13.3)		1 (2.1)		1 (3.7)	2 (6.5)	
Intermediate	13 (21)	7 (46.7)		6 (12.8)		6 (22.2)	6 (19.4)	
High	45 (73)	6 (40)		39 (83.0)		20 (74.1)	22 (71.0)	
CERAD plaque score					0.005			0.51
None	2 (3)	1 (6.7)		1 (2.1)		0	2 (6.5)	
Sparse	7 (11)	4 (26.7)		3 (6.4)		2 (7.4)	4 (12.9)	
Moderate	8 (13)	4 (26.7)		4 (8.5)		5 (18.5)	3 (9.7)	
High	45 (73)	6 (40)		39 (83.0)		20 (74.1)	22 (71.0)	
Tau Braak stage					0.015			0.74
0–II	4 (6.5)	2 (13.3)		2 (4.3)		1 (3.7)	3 (9.7)	
III–IV	12 (19.4)	6 (40.0)		6 (12.8)		5 (18.5)	6 (19.4)	
V–VI	46 (74.2)	7 (46.7)		39 (83.0)		22 (71.0)	22 (71.0)	
Alpha synuclein LBD Braak stage					0.006			*X* ^2^ = 0.57 0.45
0–III	33 (53.2)	3 (20)		30 (63.8)		13 (48.2)	18 (58.1)	
IV–IV	29 (46.8)	12 (80)		17 (36.2)		14 (51.9)	13 (42.0)	
McKeith DLB likelihood					0.011			0.74
None	24 (38.7)	2 (13.3)		22 (46.8)		9 (32.1)	14 (45.2)	
Low	14 (22.6)	3 (20)		11 (23.4)		8 (28.6)	6 (19.4)	
Intermediate	12 (19.4)	3 (20)		9 (19.2)		6 (21.4)	5 (16.1)	
High	12 (19.4)	7 (46.7)		5 (10.6)		5 (17.9)	6 (19.4)	
LBD distribution					**0.020**			0.46
None	24 (38.7)	2 (13.3)		22 (46.8)		8 (29.6)	14 (45.2)	
Brainstem	2 (3.5)	1 (6.7)		1 (2.1)		1 (3.7)	1 (3.2)	
Limbic	9 (15.5)	3 (20)		6 (12.8)		3 (11.1)	6 (19.4)	
Neocortical	21 (32.8)	9 (60)		12 (25.5)		11 (40.7)	8 (25.4)	
Amygdala	6 (10.3)	0		6 (12.8)		4 (14.8)	2 (6.5)	
TDP‐43 LATE NC					0.729			0.353
None	42 (67.7)	11 (73.3)		31 (66.0)		15 (55.6)	24 (77.4)	
Stage 1	4 (6.5)	1 (6.7)		3 (6.4)		3 (11.1)	1 (3.2)	
Stage 2	11 (17.7)	3 (20.0)		8 (17.1)		7 (25.9)	4 (12.9)	
Stage 3	5 (8.1)	0		5 (10.6)		2 (7.4)	2 (6.5)	
Vascular disease					**0.031**			0.062
None	6 (9.7)	1 (6.7)		5 (10.6)		1 (3.7)	5 (16.1)	
Mild	9 (14.5)	5 (33.3)		4 (8.5)		1 (3.7)	6 (19.4)	
Moderate	18 (29.0)	6 (40.0)		12 (25.5)		8 (29.6)	9 (29.0)	
Severe	29 (46.8)	3 (20)		26 (55.3)		17 (63.0)	11 (35.5)	
Number of co‐pathologies, mean (SD)	3.27 (1.06)	3.13 (1.36)		3.32 (0.96)	0.98	3.67 (1.04)	2.97 (1.02)	**0.004**

*Note:* LBD = McKeith ‘moderate’ or ‘high’. AD = NIA Reagan ‘high’ or in 1 case ‘intermediate’ where there was no alternative neuropathological diagnosis. Fisher's exact used when cell count < 5, otherwise chi2. Continuous non‐parametric variables assessed with Mann‐Whitney test. The bold values signify *p* values < 0.05.

Increased overall number of neurodegenerative pathologies, and separately increased severity of vascular co‐pathology, were associated with increased odds of hallucinations late in disease (vascular co‐pathology OR 2.34 [95% CI 1.18–4.67], *p* = 0.015; number of co‐pathologies OR 2.19 [95% CI 1.16–4.13], *p* = 0.016). (see Figure 4B).

### 3.5 Longitudinal Change in Hallucinations Associated With Neuropathology

In mixed effect logistic regression models adjusted for age, sex, time to death and cognition, higher densities of plaques (CERAD score) were associated with lower odds of hallucinations (*β* = −2.84, 95% CI −5.22 to −0.46, *p* = 0.019) but higher CERAD scores associated with greater increase in hallucinations over time (*β* = 0.82, 95% CI 0.08 to 1.56, *p* = 0.029). Similarly, higher Braak tau stages were associated with lower odds of clinically significant hallucinations (*β* = −1.63, 95% CI −3.04 to −0.22, *p* = 0.024) with an interaction with time (*β* = 0.45, 95% CI 0.02 to 0.88, *p* = 0.042).

Higher Braak α‐synuclein stages were associated with increased odds of hallucinations (*β* = 0.90, 95% CI 0.12 to 1.67, *p* = 0.023) and there was no interaction with time. Presence of LBD in neocortical regions (but not in the brainstem, limbic regions or amygdala) was associated with increased odds of hallucinations (*β* = 5.08, 95% CI 0.24 to 9.93, *p* = 0.040).

Increased severity of vascular pathology was associated with increasing frequency of hallucinations over time (*β* = 0.52, [95% CI 0.08–0.95], *p* = 0.021) and there was no association with stage of TDP‐43 LATE NC and risk of hallucinations.

### 3.6 Discussion

In this autopsy cohort with longitudinal characterisation of neuropsychiatric symptoms, hallucinations in early disease were more common in patients with pLBD than pAD or mixed AD+LBD. However, the trajectory of hallucinations differed in pAD and mixed AD+LBD with a greater increase in hallucinations as disease progressed such that, in the later stages of dementia, there was no difference in the odds of developing hallucinations across neuropathological groups. Moreover, the neuropathological substrates associated with hallucinations evolved over the course of disease. LBD in the neocortical regions appears to be the primary driver of early‐onset hallucinations while the aetiology of late‐onset hallucinations is more heterogeneous with additive co‐pathologies including vascular pathology and increasing density of amyloid and tau tangles implicated. The association between number co‐pathologies and hallucinations in late disease suggests that in more advanced disease where pathology is likely to be more widespread, various neuropathological substrates may contribute additively to the aetiology of hallucinations.

#### 3.6.1 Difference Across Neuropathological Diagnosis

The earlier onset of visual hallucinations in LBD relative to AD is well described and, here we also demonstrate the discriminatory potential of early‐onset hallucinations for pDLB v versus mixed AD+LBD [19, 29]. The diagnostic utility of hallucinations in discriminating pDLB declines over time because hallucinations increase in frequency in both pAD and mixed AD+LBD during the course of disease. However, previous longitudinal post‐mortem studies suggest that late‐onset of hallucinations in mixed AD+LBD may indicate heterogeneity in the temporal deposition of neuropathological substrates [19, 56, 57, 58]. In a quantitative post‐mortem study of patients with mixed AD+LBD, those with AD clinical phenotype exhibited higher burden of hyperphosphorylated tau and greater hippocampal load compared to cases with a DLB phenotype [59]. Similarly, in patients with mixed ADNC and α‐synuclein pathology, cases with clinical DLB had almost no mature tau tangles whereas those with clinical AD exhibited high levels of mature tau, which was negatively associated with core clinical features of DLB [60]. This suggests that in mixed AD+LBD with an AD clinical phenotype, the presentation is primarily driven by ADNC which likely precedes α‐synuclein deposition, while in clinical presentations of DLB, LBD appears to be the primary neurodegenerative process [59]. Indeed, a recent in vivo study found mixed pathology to be less common early in the course of disease [30]. The complex interactions between α‐synuclein, β‐amyloid, and tau pathologies and their temporal relationship remain incompletely characterised [61] but animal models suggest that α‐synuclein promotes tau aggregation, while β‐amyloid accelerates the spread of both pathologies [61, 62, 63, 64, 65]. The heterogeneity in the clinical presentation of mixed AD+LBD may be influenced by temporal differences in sequential neuropathological changes but further clinicopathological studies with in vivo biomarkers and longitudinal clinical assessments are needed to clarify how the evolution of pathologies over time influences clinical phenotypes.

However, we also demonstrate that late‐onset hallucinations occur in pAD at similar frequency to pLBD and AD+LBD, suggesting that in late disease stages, the aetiology of neuropsychiatric symptoms (NPS) is more heterogeneous and cannot reliably differentiate dementia subtypes. This late emergence of hallucinations may reflect a widespread network effect particularly as the increasing frequency of hallucinations over time in mixed AD+LBD was not independent of cognitive decline. Supporting this, patients with mixed AD+LBD exhibit alterations in functional connectivity within the default mode network that closely resemble AD, potentially contributing to differences in clinical presentation between DLB with and without AD co‐pathology [66]. Additionally, the inflammatory profile in mixed AD+LBD largely reflects AD pathology, which may also account for the psychiatric phenotype aligning more closely with AD. [67]. These factors contribute to the relatively poor diagnostic discrimination of patients with mixed AD+LBD and highlight the need for biomarkers that can capture the temporal evolution of underlying pathology [19, 22].

We did not identify differences in the clinical course of delusions, depression, agitation, apathy across neuropathological diagnoses. This may reflect the small sample size of this study, but nevertheless any subtle differences which cannot be detected in small populations are unlikely to be diagnostically useful in clinical populations on an individual level.

#### 3.6.2 Clinical Course of Hallucinations

The evolution in the aetiology of hallucinations across the course of neurodegenerative dementias may also underpin some current inconsistencies reported in the literature. Recent in vivo studies have suggested that patients with pure LBD, without AD co‐pathology, have a higher prevalence of core features of Lewy body dementia including visual hallucinations [23, 25]. This is at odds with a number of clinicopathological studies which suggest that both AD and LB pathologies contribute additively to hallucinations [11, 24]. However, cross‐sectional *post‐*mortem studies primarily capture end‐stage disease while in vivo studies often include patients soon after diagnosis [10, 11, 13]. Therefore the temporal variation in the aetiology of hallucinations we describe suggests that early in the course of disease isolated LBD drive hallucinations, while in the later stages of disease with more widespread neuropathological changes, numerous co‐pathologies may contribute additively.

Several co‐pathologies were implicated in the aetiology of late‐onset hallucinations. An association between psychosis and vascular changes has previously been reported, both in a larger post‐mortem study in late‐stage AD and between severe psychotic symptoms and advanced cerebral amyloid angiopathy and small vessel disease in a subsection of the current cohort [68, 69]. We found an increased burden of amyloid and tau was associated with lower odds of hallucinations early in disease and increasing odds of hallucinations over time. This suggests ADNC may be implicated in the development of these symptoms as they become more widespread and may underlie some of the inconsistencies in associations between NPS and amyloid‐β (Aβ) and tau pathologies in vivo PET and CSF studies [70, 71, 72, 73, 74, 75, 76, 77, 78]. Previous clinicopathological studies suggest an additive effect of neuropathological substrates on NPS and we found evidence for an increasing impact of co‐pathology as disease progressed in the later stages of disease [11, 24]. This suggests that a number of different substrates can contribute affecting networks to cause hallucinations, particularly as they become more widespread later in disease. In support of this a recent study found that visual hallucinations in AD related to the cholinergic denervation of visual processing areas in the occipital lobe rather than hypoperfusion or α‐synuclein accumulation [79]. Moreover, the phenomenology of visual hallucinations may be to some extent defined by the regions and networks involved, with the involvement of specific areas reflecting discriminant neuropathological progression and substrates [80].

#### 3.6.3 Limitations

The annual clinical assessments of NPS prior *ante‐*mortem and with neuropathological confirmation of disease is a unique feature of this study and allows track the clinical evolution of NPS associated with neuropathological change. There is also high completeness of clinical data with most attrition occurring due to death. However, to date, few patients in this cohort have both in vivo sampling and *post‐mortem* analysis and therefore this study did not involve any in vivo markers of disease. While neuropathology remains gold standard for identifying neurodegenerative change we are unable to monitor changes during earlier stages. Furthermore, the sample size of our included cohort is small and may be underpowered to detect other differences between the neuropathological groups. Therefore, while replication is needed, given the associations with hallucinations we report are supported by findings from existing studies we believe our conclusions are robust. Finally, our study did not include information related to medication which may have contributed to changes in NPS, unrelated to neuropathological changes.

### 3.7 Conclusions

Hallucinations in early disease are associated with pure LBD, while late‐onset hallucinations are common across neurodegenerative dementias and have a more heterogeneous aetiology with numerous neuropathological substrates implicated. This evolution suggests that the therapeutic target to treat hallucinations may differ both temporally and across neurodegenerative disease subtypes.

## Conflicts of Interest

DA has received research support and/or honoraria from Astra‐Zeneca, H. Lundbeck, Novartis Pharmaceuticals, Evonik, and GE Health and has served as paid consultant for H. Lundbeck, Axovant, Eisai, Heptares, Mentis Cura, Eli Lilly, and Biogen. RES has served as a paid consultant for Eisai.

## Supporting information

Supporting Information S1

## Data Availability

The data that support the findings of this study are available on request from the corresponding author. The data are not publicly available due to privacy or ethical restrictions.
