# Supplementary material for: Clinical Evolution of Neuropsychiatric Symptoms in Alzheimer's Disease and Dementia With Lewy Bodies in a Post‐Mortem Cohort
Source: Int J Geriatr Psychiatry. 2025 Apr 28;40(5):e70084. doi: 10.1002/gps.70084 (PMC12037936; doi:10.1002/gps.70084)
Supplement: Supplementary file 1 — Supporting Information S1 [file GPS-40-e70084-s001.docx]

| Outcome | Beta coefficient | 95% CI | P value |
| --- | --- | --- | --- |
| *Hallucinations* |  |  |  |
| LBD v AD | -6.66 | -11.7 − -1.64 | **0.009** |
| LBD v AD+LBD | -5.77 | -11.3 − -0.26 | **0.040** |
| Interaction with time LBD v AD | 1.48 | 0.14 − 2.82 | **0.031** |
| Interaction with time LBD v AD+LBD | 1.55 | 0.12 − 3.21 | 0.068 |
| *Delusions* |  |  |  |
| Baseline LBD v AD | -0.67 | -2.48 − 1.13 | 0.465 |
| Baseline LBD v AD+LBD | -2.27 | -4.94 − 0.39 | 0.094 |
| Interaction with time LBD v AD | 0.30 | -0.51 − 1.11 | 0.463 |
| Interaction with time LBD v AD+LBD | 0.75 | -0.43 − 1.92 | 0.212 |
| *Depression* |  |  |  |
| LBD v AD | 0.48 | −1.17 − 2.14 | 0.567 |
| LBD v AD+LBD | 0.71 | -1.26 − 2.69 | 0.479 |
| Interaction with time LBD v AD | -0.13 | -0.68 − 0.41 | 0.630 |
| Interaction with time LBD v AD+LBD | 0.12 | -0.57 − 0.80 | 0.741 |
| *Apathy* |  |  |  |
| LBD v AD | -1.00 | -2.54 − 0.54 | 0.203 |
| LBD v AD+LBD | -1.08 | -3.07 − 0.91 | 0.289 |
| Interaction with time LBD v AD | 0.40 | -0.13 − 0.93 | 0.142 |
| Interaction with time LBD v AD+LBD | 0.54 | -0.17 − 1.25 | 0.137 |
| *Agitation* |  |  |  |
| LBD v AD | -0.84 | -3.13 − 1.44 | 0.470 |
| LBD v AD+LBD | -3.43 | -7.11 − 0.25 | 0.068 |
| Interaction with time LBD v AD | -0.37 | -1.43 − 0.68 | 0.489 |
| Interaction with time LBD v AD+LBD | 0.71 | -0.88 − 2.29 | 0.382 |
| *Night-time behaviours* |  |  |  |
| LBD v AD | -1.08 | -2.91 − 0.75 | 0.246 |
| LBD v AD+LBD | 0.22 | -1.90 − 2.34 | 0.840 |
| Interaction with time LBD v AD | -0.11 | -0.77 − 0.55 | 0.738 |
| Interaction with time LBD v AD+LBD | 0.32 | -1.24 − 0.60 | 0.498 |
| *NPI>36* |  |  |  |
| LBD v AD | -1.25 | -2.86 − 0.37 | 0.132 |
| LBD v AD+LBD | -1.77 | -3.84 − 0.31 | 0.096 |
| Interaction with time LBD v AD | 0.38 | -0.29 − 1.04 | 0.269 |
| Interaction with time LBD v AD+LBD | 0.74 | -0.16 − 1.64 | 0.106 |

Supplementary Table 1. Mixed effect logistic regression models for each NPS across neuropathological diagnostic groups over time adjusted for age, sex, MMSE and time to death.

Supplementary table 2. Longitudinal associations between clinically significant hallucinations over time and neuropathological changes in mixed effects logistic regression models adjusted for age, sex, time to death and MMSE. Bold represents p values significant at the 5% level.

| Neuropathological changes | Beta coefficient | 95% CI | P value |
| --- | --- | --- | --- |
| Alpha synuclein LBD Braak stage | 0.90 | [0.12 − 1.67] | **0.023** |
| *LBD Braak * time* | -0.19 | [-0.39 – 0.00] | 0.055 |
|  |  |  |  |
| Braak tau staging | -1.63 | [-3.04 – -0.22] | **0.024** |
| *Braak tau * time* | 0.45 | [0.02 – 0.88] | **0.042** |
|  |  |  |  |
| Amyloid density CERAD | -2.84 | [-5.22 – -0.46] | **0.019** |
| *CERAD * time* | 0.82 | [0.08 – 1.56] | **0.029** |
|  |  |  |  |
| Severity of vascular disease | -1.74 | [-3.72 – 0.24] | 0.085 |
| *CVD * time* | 0.59 | [0.06 – 1.11] | **0.029** |
|  |  |  |  |
| TDP-43 LATE stage | -1.65 | [-3.87 – 0.56] | 0.143 |
| *TDP-43 * time* | 0.53 | [-0.08 – 1.15] | 0.087 |
|  |  |  |  |
| Co-pathology | -1.39 | [-3.38 – 0.61] | 0.172 |
| *Number of co-pathologies * time* | 0.51 | [-0.05 – 1.08] | 0.075 |
